# Short- and Long-Term Outcomes after Radiofrequency Ablation of Osteoid Osteomas

**DOI:** 10.3390/jpm14040401

**Published:** 2024-04-10

**Authors:** Thomas J. Vogl, Michael Bialek, Katrin Eichler, Renate Hammerstingl, John Bielfeldt, Stephan Zangos, Jan-Erik Scholtz, Hamzah Adwan

**Affiliations:** Clinic for Radiology and Nuclear Medicine, University Hospital Frankfurt, Goethe University, 60590 Frankfurt am Main, Germany; michael.bialek@gmx.de (M.B.);

**Keywords:** bone tumor, osteoid osteoma, pain, thermal ablation, percutaneous radiofrequency ablation, complications, life quality

## Abstract

The aim of this study was to evaluate treatment of osteoid osteomas using bipolar radiofrequency ablation (RFA) and patients’ quality of life before and after therapy. We retrospectively evaluated patients who underwent bipolar RFA of osteoid osteomas between 2001 and 2016. We assessed patients’ symptoms before and after treatment (four weeks after treatment and long-term) using a questionnaire including severity and quality of pain on a 10-point scale (1 = no pain, 10 = severe pain), motion restrictions, pain-related sleep disorders, and necessary pain medication. In addition, we evaluated technical success, complications, hospitalization length, and patients’ satisfaction with treatment. This study included 62 patients (43 [69.4%] males, 26.2 ± 13.2 years). Average nidus size was 5.7 ± 2.6 mm. The rate of technical success was 100%. All RFAs were performed without any complications. One patient showed a recurrence, resulting in a recurrence rate of 1.6%, which was successfully treated by another session of RFA. Average hospitalization length was 1.5 ± 0.5 days. A total of 36 patients (58.1%) participated in the questionnaire, reporting an average pain severity of 8.2 ± 1.6 before RFA compared to 3.4 ± 3.0 four weeks after and an average of 2.1 ± 2.3, 6.6 years after therapy, (both *p* < 0.001). After therapy, 31 (86.1%) patients had no pain. The majority of patients (*n* = 34, 94.4%) had reduced or absent motion restriction after therapy (*p* < 0.001). Patient satisfaction rate was 91.7%. In conclusion, bipolar RFA is a safe and effective treatment modality for osteoid osteomas and improves quality of life by reducing pain severity and motion restrictions.

## 1. Introduction

Osteoid osteomas represent the third most common noncancerous osseous tumors [1] and about 12% of bone tumors [2]. It typically occurs in the second or third decade of life [1,3], and it is more commonly seen in men than women [1]. Patients with osteoid osteomas complain about pain, especially during nighttime and show release after non-steroidal anti-inflammatory medication [1,3]. Osteoid osteomas can cause a variety of further symptoms depending on their location, such as soft tissue swelling, restricted movement, and joint effusion if it is located intra-articularly, similar to a primary articular disorder [1]. Osteoid osteomas are most commonly located in the cortex of long hollow bones, particularly in the lower extremities [1,4]. Most of them are located in the femur and tibia [4]. Furthermore, different locations such as foot or spine are possible [5], with the skull and facial bones being rarely affected [2].

There are four different locations that can be differentiated as described by Kayser et al.: subperiosteal, intracortical, endosteal, and medullary [6]. Osteoid osteomas are most commonly located intracortically, followed by medullary locations [7]. Intracortical osteoid osteomas are usually found in the diaphysis and metaphysis of the femur and tibia and medullary osteoid osteomas are often found adjacent to joints of the hands, feet, and also spine [8]. In addition to the typical clinical symptoms of patients, radiological imaging is required for the diagnosis of an osteoid osteoma. Patients with such musculoskeletal pain should be first examined by conventional X-ray images [8,9]. An osteoid osteoma appears as an oval or round radiolucent nidus, surrounded by an area of bone sclerosis [1,4]. Osteoid osteomas are small and usually have a size of 1.5–2 cm [8]. Therefore, the nidus is usually less than 1 cm in diameter and is surrounded by several centimeters of dense reactive sclerotic bone [1]. In addition to plain radiography, computed tomography (CT) imaging can accurately locate and diagnose osteoid osteomas and rule out other differential diagnoses such as chronic osteomyelitis [1,10]. Other imaging modalities like single-photon emission computed tomography and ^18^F-sodium fluoride-positron emission tomography combined with CT can also accurately diagnose an osteoid osteoma. Furthermore, ^99m^technetium-labelled bone scintigraphy can be applied for the diagnosis of osteoid osteoma by showing the typical “double density” sign, where the strongly highlighted nidus is surrounded by a less highlighted adjacent bone [7].

Magnetic resonance imaging (MRI) has been shown to be less accurate than CT in diagnosing osteoid osteoma [11,12], with a rate of 81% of correct diagnosis as benign-latent in CT images, in comparison to 19% in MRI scans [12]. In the study by Hosalkar et al. [12], lesions are often interpreted as benign-aggressive (69%) or even malignant (11%) due to the prominence of intramedullary and soft-tissue changes and therefore appear more aggressive than on the corresponding CT image [11,12,13]. Nevertheless, it is crucial to make an accurate diagnosis of osteoid osteomas on MR images because, in most cases, MRI is the next diagnostic step for children or young adults with musculoskeletal pain, to limit the exposure to ionizing radiation [14]. Certain radiologic findings, such as the half-moon sign, may aid in the diagnosis of osteoid osteomas on MRI [15]. Osteoid osteomas can spontaneously regress within a period of 6 to 15 years [16], which can be reduced to 2–3 years by using non-steroidal anti-inflammatory drugs [16]. If the pain cannot be limited with medication or if side effects arise, invasive definitive treatment is indicated [8]. Traditionally, surgery has been the preferred therapy [4], either through en-bloc resection or a burr-down technique [1,17]. The main advantage of the en-bloc resection is the most definitive removal of the complete nidus [1]. However, the bone is partially removed, which weakens it [1,4]. In recent years, minimally invasive percutaneous techniques have become the new gold standard for treating osteoid osteomas [8]. These techniques include imaging-guided percutaneous nidus removal and ablative techniques such as radiofrequency ablation (RFA), cryoablation (CA), or microwave ablation (MWA) [8].

The RFA of osteoid osteomas was initially described in a study by Rosenthal et al. in 1992 in which four patients were treated with RFA, of whom three had complete relief of symptoms [18], initially proving that the utilization of RFA can provide high rates of technical and clinical successes [19,20]. In comparison to surgery, RFA can be performed in less time and shorter duration of hospital stay [21]. It also presents a more cost-effective alternative, and results in less tissue damage and less subsequent scarring [4,10]. However, RFA for osteoid osteomas located close to nerves or blood vessels must be thoroughly planned, because of its imprecise radial energy application [4].

To further support the evidence that minimally invasive ablative techniques are safe and effective therapies for treating osteoid osteomas, we conducted a single-center retrospective study on treatment via CT-guided bipolar RFA. This study also evaluates the patients’ quality of life before and after treatment.

## 2. Materials and Methods

This retrospective, single-center study included patients who underwent CT-guided RFA of osteoid osteomas in our institution between 2001 and 2016. The local ethics committee approved this study (protocol code: 335/16 and date of approval: 27 March 2017). Inclusion criteria for RFA treatment were the clinical diagnosis of an osteoid osteoma via typical signs such as pain at night, responsiveness to non-steroidal anti-inflammatory medication as well as radiological diagnosis with conventional radiography, CT, and/or MRI.

### 2.1. Radiofrequency Ablation

**Figure 1:**
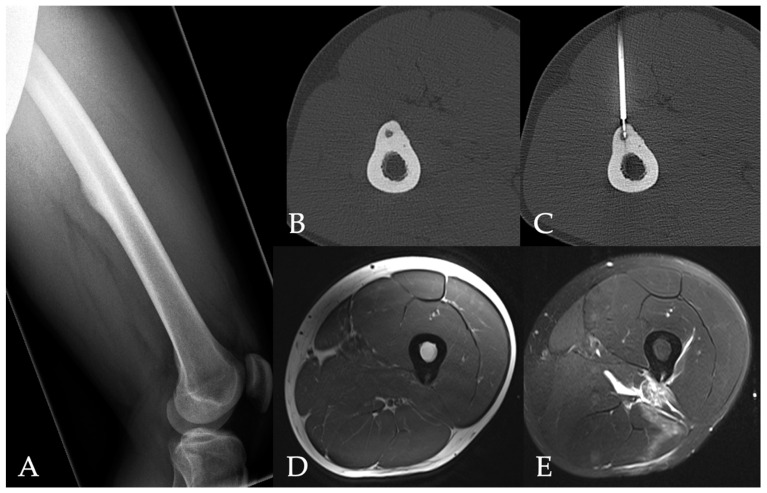
27-year-old male patient with an osteoid osteoma in the femur (**A**). Peri-interventional CT showing the lesion before and during radiofrequency ablation (**B**,**C**). Post-interventional MRI shows post-procedural changes including edema in the soft tissue without damage of the bone structure (**D** [Axial T1-weighted image], **E** [axial T2-weighted fat-saturated image]).

**Figure 2:**
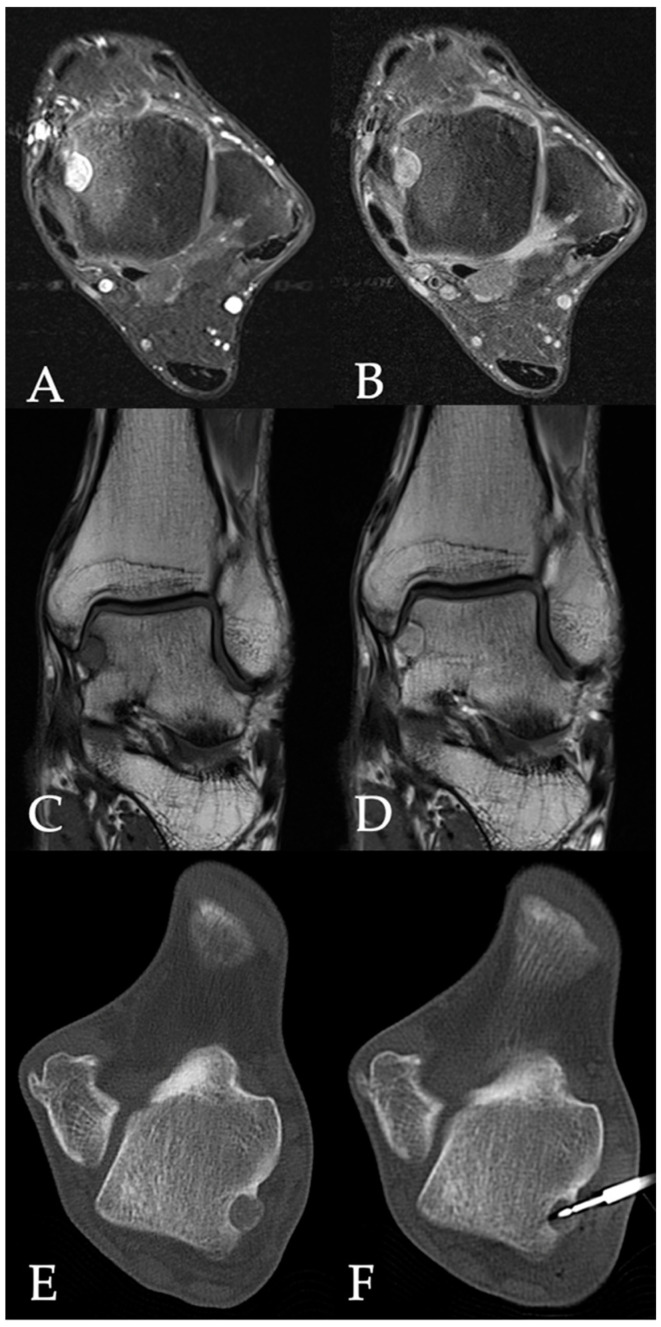
30-year-old male patient with an osteoid osteoma in the talus (**A**) [Axial proton density-weighted fat-saturated image], (**B**) [Axial contrast-enhanced T1-weighted fat-saturated image], (**C**) [Coronal T1-weighted image], (**D**) [Coronal contrast-enhanced T1-weighted image]. Peri-interventional CT images of the lesion before and during placement of radiofrequency ablation needle (**E**,**F**).

All patients underwent an unenhanced CT scan (Somatom Definition AS, Siemens Healthineers, Forchheim, Germany) of the target region to plan an access path. A bipolar coagulation electrode (Celon Pro Surge micro 150-T09, Olympus Winter & Ibe GmbH, Hamburg, Germany) was placed in the center of the nidus. Following this, the electrode was connected to a power generator (Celon Power System, Celon Lab Precision, Olympus Winter & Ibe GmbH) with an integrated automatic power control that measured tissue resistance. An alternating current field (300–500 kHz) at the tip of the electrode heated the tissue to a target temperature of 60–90 °C. The patients underwent contrast-enhanced MRI for follow-up purposes the day after RFA as well as 3 months after ablation. We noted peri- and post-interventional complications and hospitalization length. Figure 1 and Figure 2 show cases of patients with osteoid osteomas treated by CT-guided bipolar RFA.

### 2.2. Patient Questionnaire

In 2017, a patient survey was sent to all treated patients and included the following questions about symptoms prior to therapy: (1) Severity of pain using a 10-point scale (1 = no pain, 10 = most extreme imaginable pain); (2) Character of pain; (3) Appearance and duration of pain; (4) Pain-related sleep disorders; (5) Type of movement restrictions; (6) Type and intake of pain medication and its effects.

Questions after therapy included the severity of pain 4 weeks after RFA and at the time of the questionnaire (1 month–16 years after therapy). Furthermore, we asked about a possible recurrence of pain after therapy, tumor recurrence, need for pain medication, and satisfaction with the RFA therapy.

### 2.3. Data Analysis

Demographic parameter summaries were calculated for the entire subject cohort. Categorical variables were summarized using frequencies and percentages, while continuous variables were summarized as means and standard deviations. Differences in continuous variables by treatment were assessed using the Kruskal–Wallis test, and differences in categorical variables were assessed using either the Pearson χ2 test, or Fisher’s exact test. Changes in parameters pre- and post-treatment were compared using the paired *t*-test. All analyses were performed using SPSS 21.0 (IBM Corp., Armonk, NY, USA). A *p*-value of less than 0.05 was regarded as significant for all tests. RFA treatments were considered technically successful if the needle was correctly placed in the osteoid osteoma under CT-guidance and the target lesion was successfully ablated according to the treatment protocol.

## 3. Results

A total of 62 patients (43 [69.4%] males and 19 [30.6%] females) with an average age of 26.2 ± 13.2 years who underwent CT-guided RFA for their osteoid osteomas at our department were included. The majority of osteoid osteomas were localized in the lower extremities as shown in Table 1. The average nidus diameter was 5.7 ± 2.6 mm with a range from 2 to 15 mm (Table 1).

Technical success was achieved in all RFA therapies. One patient (1.6%) had a recurrence, which was successfully treated by a second RFA 5 months after initial therapy. No peri- or post-therapeutic complications were observed. The average hospitalization length was 1.5 ± 0.5 days. A total of 36 patients (58.1%) replied to the questionnaire. Most patients (*n* = 24, 66.7%) opted for RFA therapy within 24 months after the onset of pain. The rest of the patients sought RFA treatment 2 years after the pain onset. While most of the patients (*n* = 16, 44.4%) had pain during day and night, 14 (38.9%) patients reported pain only during the night, and 6 patients (16.7%) had pain only during the day. Most commonly, pain was characterized as “stinging” (*n* = 15, 41.7%) followed by “oppressive” (*n* = 10, 27.8%). Six patients at a rate of 16.7% experienced mixed pain qualities (stinging and oppressive). A total of five patients (13.8%) could not exactly describe the quality of their pain. Regarding the restriction of movement, most patients reported pain while “walking/ running” followed by pain during “standing”. The majority of patients (*n* = 34, 94.4%) had reduced or absent motion restriction after therapy (*p* < 0.001). A total of 29 patients had pain-related sleep disorders at a rate of 80.6%. The majority of patients (*n* = 30, 83.3%) required pain medication, commonly Ibuprofen and Aspirin, before the treatment. Pain was relieved by this medication in 27 (90.0%) patients. Characteristics and management of pain are summarized in Table 2.

**Figure 3:**
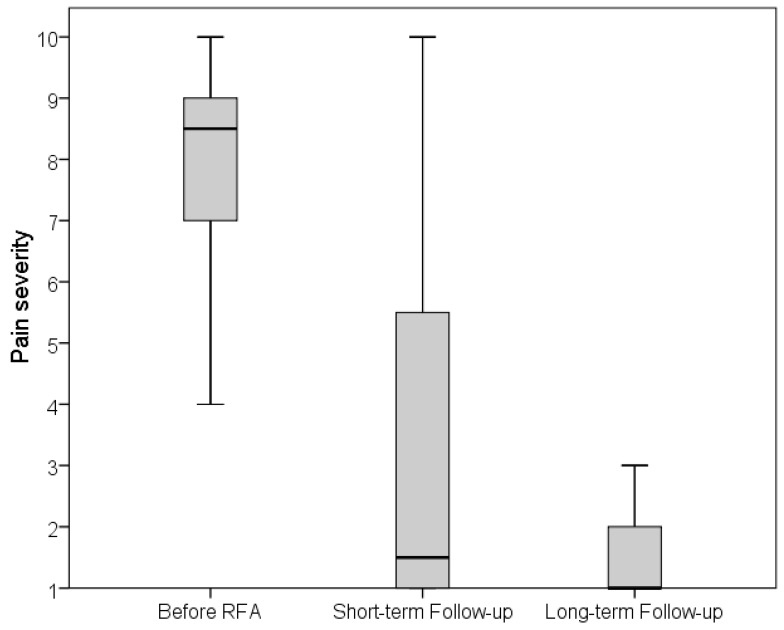
Intensity of pain (1 = no pain, 10 = worst imaginable pain) before, short-term (4 weeks), and long-term, after radiofrequency ablation (RFA).

All patients reported pain prior to therapy with an average pain severity of 8.2 ± 1.6 on the pain scale. Four weeks after RFA, half of the patients (*n* = 18, 50%) did not have pain and the other half (*n* = 18, 50%) reported an average pain severity of 3.4 ± 3.0. In the long-term follow-up after an average of 80 ± 45 months, the majority of patients (*n* = 25, 69.4%) did not have pain, while 11 patients (30.6%) reported an average pain intensity of 2.1 ± 2.3 (Figure 3). Thus, pain severity was significantly reduced 4 weeks after RFA and also in the long-term follow-up compared to before the RFA (*p* < 0.001). Intraindividual comparison also showed a significant reduction in pain severity within 4 weeks and in the long-term after therapy compared to prior RFA (both *p* < 0.001). Overall, almost all patients (*n* = 33, 91.7%) were satisfied with RFA therapy.

## 4. Discussion

In this retrospective study of CT-guided bipolar RFA of osteoid osteomas we could show a beneficial short- and long-term outcome in patients’ quality of life compared to prior to RFA therapy.

Our study could show excellent results with patients having a significant short- and long-term reduction in pain after RFA therapy as well as a high satisfaction rate of 91.7%. The majority of patients also had a reduced or absent motion restriction after therapy and the pain reduction was significant in short- and long-terms after therapy compared to prior RFA.

The most common location of osteoid osteomas in our population was in the long bones of the lower extremities, mostly femur and tibia. In concordance with the literature [22,23,24], we achieved technical success in all performed RFA therapies, showing that RFA is a very suitable therapy option for osteoid osteoma. An advantage of performing RFA is that only a small access to reach the osteoid osteoma is necessary, which results in a better preservation of surrounding bone mass [19], in comparison to surgical treatments, especially if an en-bloc resection for the osteoid osteoma is being carried out [1]. Another advantage is the cost effectiveness of RFA, since no prolonged hospitalizations, or extensive surgical equipment is necessary. Yu et al. compared percutaneous CT-guided RFA with operative treatment for spinal osteoid osteomas in 28 patients [25]. They showed that RFA was performed significantly faster than surgery and that the patients in the ablation group had significantly shorter hospitalization time as well as less loss of blood than surgically treated patients. The complication rate was also lower in the RFA group compared to the surgical group. Sangiorgio et al. could also show that RFA has a lower complication rate of 4.4% in comparison to surgical excision with 7.8% [26]. Though, the recurrence rate was slightly higher in the RFA group, with 6.7% [26].

Göksel et al. compared RFA with curettage in treating a total of 24 children with osteoid osteomas [21]. The recurrence rate was 18.1% (2/11) in the curettage group and 15.3% (2/13) in the RFA group. However, in our study the rate of recurrence was significantly lower with 1.6% (1/62). Baal et al. could show in their study that some factors such as tumor length, female gender, and age ≤13 years were significantly associated with recurrence of osteoid osteomas [27]; therefore, complete ablation of the total tumor mass must be ensured in these patients. Furthermore, in a meta-analysis by Efthymiadis et al. regarding osteoid osteoma of the hip, the authors propose RFA as a first-line treatment in comparison to percutaneous resection drilling and arthroscopy, due to its success rate of above 98% and its relatively low complication rate, although two iatrogenic femur fractures were reported [28]. Therefore, surgical excision may result in similar recurrence rates as RFA, though on the other hand, RFA has lower complication rates and is performed in a shorter period of time [21,25,26].

Regarding different ablative techniques, a systematic review and meta-analysis by Shanmugasundaram et al. evaluated a total of 1528 patients who underwent ablative treatments for osteoid osteomas including RFA, MWA, CA, and laser ablation [29]. Most of the included patients were treated by RFA (*n* = 1133). The rates of technical success ranged from 84% to 97.8% and rates of clinical success from 94.2% to 100%. The rate of recurrence ranged from 0% to 5.79%, although RFA had the second highest recurrence rate after laser ablation, no statistically significant difference between the different ablation modalities could be shown. The rates of minor complications ranged from 2.12% to 16% and rates of major complications from 0% to 0.79%, a statistically significant difference could also not be shown. Similar results were seen in a study by Lindquester et al. comparing RFA and CA [30]. Both studies could show the high efficacy and safety of ablative treatments for osteoid osteomas, no matter which type of ablative treatment is chosen, therefore enabling an interchangeability of ablation therapies depending on availability for one’s institution [29,30]. RFA of osteoid osteomas has a low rate of complications, nonetheless there are still various complications that may happen. These complications include infections, burns, hematomas, and fracture of materials for example needles or drills [31,32]. Further complications such as fractures of bones, injuries of adjacent blood vessels or nerves may also occur [32]. In our study, we did not observe any complications in all performed RFA sessions, showing that RFA is very safe, although we only treated a small number of patients. As mentioned, another main advantage of local ablation therapy such as RFA is the very short hospitalization length. In our patient cohort the average hospitalization was 1.5 days, which was similar to other studies [33,34].

## 5. Limitations

This single-center study had several limitations which need to be addressed. Firstly, the study was limited by potential recall bias and due to its retrospective design. Secondly, similar to studies from other authors, the number of treated patients was low with only 62 patients throughout 15 years. Furthermore, we received a response to our questionnaire from only 36 patients out of 62 patients. Finally, there was no control group in the current study to accurately verify the efficacy and safety of RFA in comparison to other treatments such as surgery or other minimally invasive percutaneous treatments for osteoid osteomas.

## 6. Conclusions

This study shows that CT-guided bipolar RFA is a safe and effective modality for the treatment of osteoid osteomas and improves quality of life by reducing pain severity and motion restrictions. RFA can be performed with a minimum hospitalization time and with high patient satisfaction.

## Figures and Tables

**Table 1:** Patient characteristics.

Parameter	Patients *n* = 62
Age, y	26.2 ± 13.2
sex	
Female	19 (30.6%)
Male	43 (69.4%)
Tumor localization	
Femur	27 (42.9%)
Tibia	19 (30.2%)
Humerus	4 (6.3%)
Spine	4 (6.3%)
Cervical	1 (1.6%)
Thoracic	1 (1.6%)
Lumbar	2 (3.2%)
Talus	3 (4.8%)
Ilium	2 (3.2%)
Fibula	1 (1.6%)
Rib	1 (1.6%)
Thumb	1 (1.6%)
Index finger	1 (1.6%)
Nidus size, mm	5.7 ± 2.6

Note. Values are *n* (%) and mean ± standard deviation.

**Table 2:** Characteristics and management of pain.

Parameter	Patients *n* = 36
Pain quality	
Stinging	15 (41.7%)
Oppressive	10 (27.8%)
Stinging and oppressive (mixed)	6 (16.7%)
Indescribable	5 (13.8%)
Time of pain	
All-day	16 (44.4%)
Nighttime	14 (38.9%)
Daytime	6 (16.7%)
Pain-related sleep disorders	29 (80.6%)
Pain medication	
Yes	30 (83.3%)
Pain relief	
Yes	27 (90%)
No	3 (10%)

Note. Values are *n* (%).

## Data Availability

The data presented in this study are available on request from the corresponding author. All requests must be justified and will be checked according to privacy and possible ethical restrictions.
